# Factors Impacting Mobile Health Adoption for Depression Care and Support by Adolescent Mothers in Nigeria: Preliminary Focus Group Study

**DOI:** 10.2196/42406

**Published:** 2025-04-09

**Authors:** Lola Kola, Tobi Fatodu, Manasseh Kola, Bisola A Olayemi, Adeyinka O Adefolarin, Simpa Dania, Manasi Kumar, Dror Ben-Zeev

**Affiliations:** 1 WHO Collaborating Centre for Research and Training in Mental Health, Neurosciences and Drug and Alcohol Abuse, Department of Psychiatry College of Medicine University of Ibadan Ibadan Nigeria; 2 Centre for Global Mental Health Kings College London London United Kingdom; 3 School of Computer Science University of Leicester Leicester United Kingdom; 4 Department of Health Promotion and Education College of Medicine, Faculty of Public Health University of Ibadan Ibadan Nigeria; 5 Association for Reproductive and Family Health (ARFH) Ibadan Nigeria; 6 Department of Population Health School of Medicine, Institute for Excellence in Health Equity New York University New York, NY United States; 7 Department of Psychiatry and Behavioral Sciences BRiTE Center University of Washington Seattle, WA United States

**Keywords:** adolescent perinatal depression, primary care, mHealth app, user centered design, smartphone, human-centered design, HCD, depression, postpartum, perinatal, postnatal, teenage, adolescent, youth, low-middle-income countries, LMIC, middle income, adoption, acceptability, mobile health, mHealth, mobile app, women’s health, mental health, depressive

## Abstract

**Background:**

Mobile health (mHealth), the use of mobile technology in health care, is increasingly being used for mental health service delivery even in low- and middle-income countries to scale up treatment, and a variety of evidence supports their potential in different populations.

**Objective:**

This study aims to use the Social Cognitive Theory (SCT) as a lens to explain knowledge of mHealth use for mental health care, personal behavioral capabilities, and the external social contexts that can impact the adoption of an mHealth app for depression care among perinatal adolescents in Nigeria.

**Methods:**

At the preliminary stage of a user-centered design (UCD), 4 focus group discussions were conducted among 39 participants: 19 perinatal adolescents with a history of depression and 20 primary care providers. Guided by the SCT, a popular model used for predicting and explaining health behaviors, we documented participants’ knowledge of mHealth use for health purposes, advantages, and challenges to the adoption of an mHealth app by young mothers, and approaches to mitigate challenges. Data collection and analysis was an iterative process until saturation of all topic areas was reached.

**Results:**

The mean age for young mothers was 17.3 (SD 0.9) years and 48 (SD 5.8) years for care providers. Mistrust from relatives on mobile phone use for therapeutic purposes, avoidance of clinic appointments, and sharing of application contents with friends were some challenges to adoption identified in the study population. Supportive personal factors and expressions of self-efficacy on mobile app use were found to be insufficient for adoption. This is because there are social complications and disapprovals that come along with getting pregnant at a young age. Adequate engagement of parents, guardians, and partners on mHealth solutions by care providers was identified as necessary to the uptake of digital tools for mental health care in this population.

**Conclusions:**

The SCT guided the interpretations of the study findings. Young mothers expressed excitement at the use of mHealth technology to manage perinatal depression. Real-life challenges, however, need to be attended to for successful implementation of such interventions. Communications between care providers and patients’ relatives on the therapeutic use of mHealth are vital to the success of a mHealth mental health management plan for depression in young mothers in Nigeria.

## Introduction

The World Health Organization (WHO) strongly recommends the rapid widespread use of technology as a possible solution for transforming pressing public health services [1]. Mobile Health (mHealth), the use of mobile technology in health care, is increasingly being used for mental health service delivery even in low- and middle-income countries to scale up treatment, and a variety of evidence supports their potential in different populations [2]. Mobile phones are ubiquitous globally and in low-income countries with evidence of use in different population groups [3]. Recent reports indicate their high use among the youth perinatal population in Nigeria [4]. In Nigeria, digital research has been successfully conducted for perinatal mental care within the WHO Mental Health Global Action Programme (mhGAP) task-shifting initiative in primary care [4-6] to address barriers to care in patients. Task shifting, which is a process of delegating tasks to less specialized health care workers where appropriate, is recommended by the WHO to bridge the treatment gap in global mental health [7]. Digital health care presents avenues to further scale-up care in the process of task shifting [1]. Technology-based studies for perinatal care in Nigeria have been implemented to increase pregnant women’s access to care, but several knowledge gaps exist on factors that influence the use and adoption of mHealth in adolescent mothers [8,9]. Addressing these gaps is crucial for increasing access in a vulnerable population. Perinatal adolescents, often unmarried, might have their pregnancies disputed by the father of their child and could also have little or no social support from family members [8,9] necessitating the need for age and context-specific health care for this vulnerable population.

Perinatal depression, a condition common in pregnant women, is higher among adolescent mothers than older mothers [10,11] occurring during pregnancy and up to one year after childbirth. Untreated perinatal depression is a risk for negative health outcomes for mothers and their infants [12]. There are limited resources for managing mental disorders in primary care in low-income countries [13] and access for young mothers is even more limited by multiple age-related access barriers, which often translate to self and social stigma [8]. The complexities of hindrances to care experienced by this population indicate the need for practical and flexible interventions tailored to meet their unique needs [14].

Mobile phone apps, which are software programs designed to work on mobile devices, can serve as mHealth tools used in real-time for mental health care [15,16]. Mobile phone apps offer easy and affordable digital access to mental health information and solutions and can be used to increase internet-based access to mental health services. Evidence supports the involvement of users in the designing and developing of technology-based interventions to increase their potential for success and uptake [17]. User-centered designs [18] allow users to participate in iterative developments and design processes of mHealth tools, for reflection of users’ needs in treatment designs. The use of mHealth even in low-resource settings [2] and the willingness of young mothers to engage in mobile phone use for health care services [4] present several opportunities for the design of technology-based interventions that attend to their unique needs [19].

In this study, we explored the benefits, barriers, and mitigating factors to mHealth adoption for depression by adolescent mothers in Nigeria through a theoretical lens. Theories play a crucial role in explaining health behaviors, and in digital health care they have provided frameworks for predicting and influencing human behaviors in the context of health and well-being. [20,21]. In this study, however, we used the Social Cognitive Theory (SCT), a widely recognized theory of behavior change [22] in explaining and understanding participants’ knowledge of mHealth use for mental health care (personal experiences), personal behavioral capabilities of adolescent mothers to engage with a mobile phone app for depression care (behavioral factors), and external social context (environmental factors) that may impact their expected health outcomes [23].

Focus group discussions (FGDs) were carried out with adolescent mothers as part of the second stage of a user-centered design (UCD) of a mHealth app that was designed to complement face-to-face care for perinatal depression within routine primary care. The question guide explored participants’ knowledge of mHealth use, the perceived advantages and challenges of application use, and approaches to mitigate identified challenges. The research questions were as follows:

1. What is the knowledge of mHealth use for mental health care among young mothers and care providers in primary care?
2. What are the potential barriers and facilitators of mobile app adoption in this population? What challenges could limit its use?
3. What are the possible strategies to mitigate the challenges to mHealth apps adoption by young mothers?

## Methods

### Participants and Study Setting

The study was conducted in Ibadan, southwest Nigeria using FGDs: 2 FGDs of young mothers (n=19), and 2 separate ones among care providers (n=20). The venue was a seminar room at the Department of Psychiatry, University of Ibadan that was chosen because it was private and easily accessible using public transportation. All 4 FGDs took place in a 2-week period in February 2020. Participants were recruited from the database of an ongoing trial on perinatal depression in primary care [24]. A total of 30 participants aged between 16 and 19 years had completed treatment in an ongoing trial (Responding to the challenge of Adolescent Perinatal Depression [RAPiD]) [24], and those in remission from clinical depression as indicated by their hospital records were selected. Only 22 of them had phone numbers that rang through on the mobile network. The phone numbers of the remaining 8 were no longer available on the mobile networks. Even though all 22 young mothers who were contacted by phone indicated their availability for the FGDs, 3 participants later called to indicate their unavailability on the days chosen for the discussions, leaving 19 participants who attended the FGDs. On the part of the care providers, 20 primary care providers from the same trial’s intervention arm were invited, and all accepted and participated in the FGDs. All participants were briefed on the purpose of the FGDs when they were contacted on the phone. The young mothers and the clinicians were selected from clinics in the 11 local government areas in Ibadan. On the days of the FGDs, the facilitator gave additional detailed information on the purpose of the FGDs. All participants signed an informed consent form after giving verbal consent to participate in the study. In total, 3 groups had 10 participants each, while the fourth (a young mother’s group) had 9 participants.

### FGD Procedure

The average duration for the FGDs was 90 minutes. Author LK, a medical sociologist, moderated all 4 groups using predetermined open-ended questions. Author TF was the timekeeper and note-taker in all the discussions. Both LK and DA had no previous contact or interactions with study participants. Following the introductions, the facilitator asked the initial question on knowledge of mobile phone use for health purposes. Participants who indicated such knowledge were asked to describe the interventions they were aware of. The facilitator probed on the mode of delivery of such interventions identified, the information content, and the disease conditions they were used for. Participants were then asked about their familiarity with mobile phone apps, and irrespective of the answer given, the facilitator showed all participants a video-based mobile phone app on an Infinix Hot 7 Pro Android phone. This demonstration ensured that participants understood what an mHealth app was before asking them related questions. All FGDs were audiotaped (for research purposes only), transcribed verbatim, and analyzed shortly after they were completed. Therefore, data collection and analysis became an iterative process until saturation of all topic areas was reached. Responses of participants were deidentified, and the transcribed data was not shared with anyone outside the investigating team.

### Data Analysis

The data were transcribed by 2 experienced transcriptionists TA (a research assistant) and DA (co-author). Authors LK and DA conducted thematic coding of the transcripts manually along with the areas of interest. Specifically, LK created the initial code, which the authors reviewed. Identical subcodes were merged while redundant ones were removed. The interview transcripts were then read to understand and familiarize by LK and DA and independently coded to highlight texts relevant to the derived themes. The process led to a descriptive account focused on identifying critical variables for each heading [25].

SCT was used to explain the interpretations of positive supports for behavior changes in areas of personal, behavioral, and environmental factors [26]. In this investigation, personal factors (eg, previous knowledge of mHealth risks and benefits) were identified as prerequisites for behavior change but not the sole factor for the needed changes. Also, given the current dearth of mHealth use in mental health care in Nigeria and in the study population of young mothers, the point of inquiry on previous knowledge of use focused on all known areas of use within general health care. Second, the behavioral capability of adolescent mothers, their beliefs in their ability (self-efficacy) to engage in mobile phone app use for health purposes to carry out desired actions and produce desired effects, was assessed within the present social context on the roles and autonomy of adolescent girls as foundational to their likelihood of adopting and maintaining mHealth use [27,28]. Third, we documented outcome expectancies of engaging in mobile app use through participants’ perceptions of the advantages and disadvantages of such action with the aim of identifying motivating and mitigating factors to assist in achieving desired results [29].

### Ethical Considerations

The University College Hospital, University of Ibadan, ethical review board approved the study in accordance with the 1964 Declaration of Helsinki and its later amendments (UI/EC/19/0165). In this study, only perinatal adolescents aged 16-19 years who could give consent to participate, and primary health care providers over the age of 18 years were recruited. Study data from observations and audio recordings during the FGDs were stored as an mp4 file on a password-protected encrypted computer at the Department of Psychiatry, University of Ibadan. Group facilitators took notes that strictly contained only deidentified information. All the participants approached were given full information on study purposes and procedures and told that their participation was voluntary, before they were invited to participate. The informed consent form had two original copies for those who agreed to participate: one of these was stored securely in the research file and a copy was given to the participant. All participants gave voluntary responses to interview questions and were told they could decline to answer any questions that they chose. The adolescent girls received a total sitting and transport allowance of the equivalent of US $10, while the primary care providers received US $15.

## Results

The mean age for young mothers was 17.3 (SD 0.9) years; they were predominantly single (14/19, 73.7%) with a few full cohabiting (4/19, 21.1%). The age of mothers who had some high school education was 10.6 (SD 1.9) years: 8 out of 19 (42.1%) of them were still students, 2 out of 19 (10.5%) of these were first-year university undergraduates); and 11 out of 19 (57.8%) others had dropped out of school and engaged in petty trading. The care providers were middle-aged (mean 48, SD 5.8 years) majorly female (18/19, 95%), and with significant (mean 22.2, SD 4.7 years) work experience. A summary of the demographic information of participants is presented in Tables 1 and 2. Our open-ended question guide unveiled several responses according to the themes presented in Tables 1 and 2 below.

**Table 1:** Demographic information of women participants.

Variables			Values
**Marital status, n (%)**			
	Single	14 (74)	
	Married	1 (5)	
	Cohabiting	44 (21)	
**Occupation, n (%)**			
	Students	8 (42)	
	Petty trading	11 (58)	
Age of perinatal adolescent participants (year), mean (SD)			17.3 (0.9)
Years of education of adolescent participants, mean (SD)			10.6 (1.9)

**Table 2:** Demographic information of care provider participants.

Variable			Values
**Sex of care providers, n (%)**			
	Male	1 (5)	
	Female	19 (95)	
	Total	20 (100)	
**Marital status, n (%)**			
	Married	20 (100)	
Age of care provider participants (year), mean (SD)			48 (5.8)
Years of work experience, mean (SD)			22.2 (4.7)

### Factors Related to mHealth Use

The perinatal adolescent groups had knowledge of mHealth use for health interventions in reproductive health care and mental health care. The latter knowledge was related to their participation in the ongoing RAPID study [24] and its mHealth component. Group members commonly reported receiving SMS on reproductive health promotion and prevention programs:

I receive regular text messages telling me about contraceptive use and different types

I know about text messages advising people to space their children

I don’t know who sends it but I get regular messages in Yoruba (a language in Nigeria) telling me to visit my nearest clinic to ask about family planning methods.

However, the adolescent mothers were of two minds about the usefulness of health information (SMS) in reproductive care. On the one hand, they saw it as not useful since stigma would prevent them from taking advantage of such a program because of their age:

I would be too self-conscious to go and ask for such services, also I don’t think health care providers will attend to people unmarried especially young girls who want contraceptives.

On the other hand, some agreed that the SMS provided useful information that was relevant for the future.

The majority in the perinatal adolescent groups identified their sources of knowledge of mHealth use for mental health care as through mobile phone calls or SMS for follow-up from care providers during their participation in the ongoing RAPID project [24]:

Matron would call me to remind me about clinic appointments for depression care on the day before the appointment dates.

The nurse sent me a message to remind me to take my baby for immunisation because when she called the day before then I had complained of not having much energy.

I got a call from the nurse…she asked how I was doing, the calls from her always lifts my mood.

Matron called me the first evening I visited the clinic..she said she wanted to be sure I was okay…she also told me not to forget the homework she gave me as part of the treatment for my depression.

All the care providers reported their use of mobile phone apps and SMS in various ways in their practice in maternal and child health care:

…we use a mobile app given to use by the WHO for child immunisation data cordination and we also have other one from a project on family planning which helps us in self learning.

All of the care providers had wide experiences in using phone-to-phone calls for service provision and the WhatsApp (Meta) platform for supervision and support of health interventions.

We call doctors and matrons who are our clinical supervisors on the mobile phone when needed and staff members also chat on group Whatsapp platforms for work purposes.

### Perinatal Adolescents and Mobile App Use

Both groups of participants (perinatal adolescents and primary health care providers) had positive comments on the appropriateness of mobile app use in the perinatal adolescent population for depression care, and the abilities of this population to engage its use for depression care. One care provider commented:

…you know that young people like to explore phones so delivering health services to them through this avenue increases their likelihood of engaging in such care. More than nine out of ten of these girls can read and write so accessing health care on the mobile phone is what they will be able to do.

Many of the young mothers noted it could help them to reduce the times they might have to go to the clinics because of the long waiting times they often experience:

Many times at the clinic, I get tired and just want to go home because the crowd can be many…so this kind of app, will help someone like me since it could reduce clinic visits.

A few saw it as a way to overcome access stigma related to being pregnant at a young age and cost-related barriers to health care.

Going to the clinic when I was pregnant was always troubling for me because I felt so self-conscious of my being young compared to adult mothers… so I skipped many clinic days.

Majority of the young mothers were excited about the thoughts of a mobile phone app with short videos for depression self-care, and a few also believed that it could be used by young mothers to improve users’ moods and to prevent boredom. They generally found the use of video for treatment on the application very appealing, noting that it will help them recollect their discussions with the nurses at the clinic, which would help consolidate the actions they need to improve mental health:

It will keep me from being bored (shows excitement)…I like the idea because videos will be remembered easily…and If I have such app, I can also use it to remember anything I forget that the nurse said

I watch short videos on face book, so such an app like this will be easy for me.

Many young mothers reported watching short video clips on Facebook (Meta) for leisure. Care providers also agreed that

…a health app would help reinforce treatment from the clinics and help young mothers with self-care.

Reports of both groups on mobile app use within the social context of perinatal adolescents gave very strong indications of the need to get parents, guardians, and partners to be on board and be supportive of its use. The care providers said:

Some parents might think the young mothers are using their phone as an excuse not to help with housework rather than for accessing their depression treatment.

In the same vein, young mothers’ perceptions were similar:

If my mother thinks I am staying on the phone too much and texting my boyfriend, she controls the use of my phone

…if I have an app for treatment, my mother would need to be told from the clinic for her because if she sees me staying long on the phone, she could think that I am just playing games and she can seize the phone for that reason.

One care provider had concerns about cases of severe depression that could result in functional limitations, thereby impacting the ability to use the application:

…a few might be too depressed to even use the app or even spend time looking at the treatment on it.

### Outcome Expectations of Application Adoption Among Young Mothers

In general, the two participant groups made constant reference to the positive outcome expectancies associated with engaging in mobile app use for the management of depression in this population. Some members in the perinatal adolescent groups showed visible excitement at the possibility of accessing health care through audiovisual materials on a mobile phone app by talking in high-pitched voices:

if I has an app like that when I was sick, I would have gotten better quicker because this would have increase the treatment I received.

An app with videos as treatment will be good… it could help with feelings of sadness and how to take any needed action.

I like watching videos on Facebook…they helped me when I felt down, so this is good because it will help to prevent boredom and sadness.

Although the care providers were generally positive on the outcome expectation of application use on the mental health of perinatal adolescents, 2 of the older nurses were skeptical, given the possibility of increased treatment default rates [11] among young mothers who replace clinic appointments with application use:

…if the app treatment is not properly presented to patients as what they should use along with the face-to-face clinic visits, some might not come as they should.

This will be more so if a treatment plan for depression modify clinic visits inconsideration for frequency of application use.

You know adolescents don’t like going to the clinic for appointments, my fear is that they will now not come at all if they have an app for self care.

Also, another care provider pointed out the possibility of worsening symptoms if patients are unable to use the application, for example, as a result of severe depression. Some care providers have negative consequences of application misuse if patients share treatment contents with people with similar symptoms*:*

The patients would need to be warned against sharing the app with friends or relatives with similar symptoms to avoid severe consequences.

### Mitigating Challenges to mHealth Adoption in Young Mothers

Participants identified 5 action plans to mitigate challenges to young mothers’ mHealth use. Both groups of participants agreed on the need for care providers to educate patient’s relatives on its usefulness in young mothers’ depression care:

If the nurse tells my mother that a mobile phone app is for treatment purposes, she will even be the one to encourage me to use it more so I can get well quickly.

My grandmother that I live with will cooperate with whatever they say from the clinic regarding my health.

The care providers generally agreed that mHealth use should be included as part of the total management plan for perinatal depression to remove possible misconceptions from patients who many see as an alternative to face-to-face care. Care providers and young mothers also suggested the use of phone calls from care providers to encourage and remind young mothers of application use. The majority of the care providers identified the need to warn adolescent mothers on a regular basis about the harms that could result from sharing the mHealth app with relatives and friends with similar symptoms of depression. A few of the care providers pointed out it would be necessary to block young mothers’ access to mHealth apps after treatment to guide against misuse. Both caregiver and young mother FGD groups wanted the application attractive and interactive in order to engage young mothers and encourage usage.

## Discussion

### Principal Findings

Interventions based on behavioral theory use have proven to be more effective than theoretical approaches [30,31] allowing for a deeper understanding of the subjective dimension of the process [32].

To our knowledge, this is the first UCD approach assessing the feasibility of mobile app-based depression intervention for adolescent mothers, while also identifying factors to mitigate possible challenges to use by young mothers in Africa. Our study population (mean 17.3, SD 0.9 years) was adequately educated (mean 10.6, SD 1.9) contrary to findings in previous studies where perinatal adolescents were mostly uneducated [33,34].

On the whole, young mothers’ and health workers responded positively to a video-based mHealth app used to manage adolescent perinatal depression [35]. This response could be an inference that there may be a high rate of adoption of such interventions in the future. A significant strength of the study is the participation of key stakeholders who were part of a recent trial. Contrary to a previous report on insufficient knowledge of mHealth use among clinicians in Nigeria [36], this study’s primary health care providers were knowledgeable and currently engaged in mHealth use for different health purposes.

Perinatal adolescents had a sense of personal agency (self-efficacy) with respect to using the mHealth app, which was indicative of them having the skills and abilities necessary for performing the behavior under a variety of circumstances. This self-efficacy in young mothers is ultimately linked to their general level of education in young people in Nigeria, which is a result of basic universal education in the country [37].

Young mothers and care providers noted several positive outcome expectations as advantages of application use. Both groups agreed that this mode of digital health service provision could keep young mothers in contact with care as they navigate personal- and clinical-level barriers to care [38]. The general response supported the enthusiasm of young people for technology, which increases the likelihood of their engagement with it and its usefulness for depression care in the group. This finding is supported by a recent survey of the current use of mobile phones among young people in Nigeria, which reported the broad willingness of young mothers to engage in technology use for mental health care [4]. Based on the values and expectations of the young mothers’ who reported watching videos on Facebook to entertain themselves, the idea of a video-based application therefore presented something familiar with their current pattern of entertainment, thereby increasing the likelihood of future use.

The young mothers identified the influence of social support in mediating behavior change [39,40] identifying both parents, partners, and health care providers as key stakeholders [24] in a mHealth intervention design for this population with depression. The importance of warning young mothers of the dangers of application sharing and uninstalling the mobile app after completion of treatment were potential ways to prevent misuse of mobile apps by patients. As in previous findings, the risks associated with patients sharing electronic health information with other users were an identified challenge [41].

### Study Finding Through an SCT Lens

Unlike other cognitive development theories, SCT incorporates a 3-way relationship between individuals, their self-regulating process of efficacy, and their social circumstances. In this study, we understand that supportive personal factors and the ability to engage in application use for depression care are insufficient for adoption given the limitations that can occur on the social level. Misunderstanding from parents and partners of its purposes for entertainment rather than for therapy is a main hindrance. This might be more so the case if adolescent mothers spend time away from participating in household activities due to mHealth use, or if parents suspect it is being used for other reasons such as “texting of boyfriend” or “playing games.” This necessitated the need for care providers to adequately engage parents, guardians, and partners on the health benefits of mHealth solutions for mental health care and to prepare them for assisting young mothers with future challenges that they might encounter in its use. Such applications in the future also can entail tools for health care providers, parents, and partners, which can help mitigate clinical- and family-level stresses. A combined intervention strategy of mHealth and face-to-face care is a model that provides an opportunity to reduce parents’ misconceptions about application use through interactions between care providers and patients’ relatives.

### Limitations

Data were collected from participants who were part of an ongoing clinical trial on perinatal depression that used mHealth and their current knowledge might have influenced their responses. For this reason, this study result might not be generalizable to views of all care providers or perinatal adolescents. Also, the transferability of the study finding is limited by the fact that young mothers in low-resource settings are majorly students or low-income earners and therefore might not all be able to afford smartphones if after a trial stage the intervention is found to be successful. Despite these limitations, this study provides useful information on how to mitigate identified barriers to adopting an mHealth app for adolescent perinatal depression in primary care.

### Conclusions

Young mothers expressed excitement at the use of mHealth technology to manage perinatal depression. Real-life challenges, however, need to be attended to for successful implementation of such interventions. We found that care providers and relatives of young mothers are vital stakeholders in the success of a mHealth mental health management plan to manage depression in young mothers. Our results reinforce the need for management plans that combine face-to-face care in adopting mHealth in young mothers’ mental health interventions.

## Abbreviations

FGD: focus group discussions
mHealth: mobile health
mhGAP: Mental Health Global Action Programme
RAPiD: Responding to the challenge of Adolescent Perinatal Depression
SCT: Social Cognitive Theory
UCD: user-centered design
WHO: World Health Organization
